# Comparing the Income Elasticity of Health Spending in Middle-Income and High-Income Countries: The Role of Financial Protection

**DOI:** 10.15171/ijhpm.2017.83

**Published:** 2017-07-19

**Authors:** Arturo Vargas Bustamante, Sandhya V. Shimoga

**Affiliations:** ^1^Department of Health Policy and Management, Fielding School of Public Health, University of California, Los Angeles, CA, USA.; ^2^Department of Health Care Administration, California State University, Long Beach, CA, USA.

**Keywords:** Healthcare Spending, Emerging Markets, Panel Analysis, Income Elasticity of Health Spending, Financial Protection, Middle-Income Countries

## Abstract

**Background:** As middle-income countries become more affluent, economically sophisticated and productive, health expenditure patterns are likely to change. Other socio-demographic and political changes that accompany rapid economic growth are also likely to influence health spending and financial protection.

**Methods:** This study investigates the relationship between growth on per-capita healthcare expenditure and gross domestic product (GDP) in a group of 27 large middle-income economies and compares findings with those of 24 high-income economies from the Organization for Economic Cooperation and Development (OECD) group. This comparison uses national accounts data from 1995-2014. We hypothesize that the aggregated income elasticity of health expenditure in middle-income countries would be less than one (meaning healthcare is a normal good). An initial exploratory analysis tests between fixed-effects and random-effects model specifications. A fixed-effects model with time-fixed effects is implemented to assess the relationship between the two measures. Unit root, Hausman and serial correlation tests are conducted to determine model fit. Additional explanatory variables are introduced in different model specifications to test the robustness of our regression results. We include the out-of-pocket (OOP) share of health spending in each model to study the potential role of financial protection in our sample of high- and middle-income countries. The first-difference of study variables is implemented to address non-stationarity and cointegration properties.

**Results:** The elasticity of per-capita health expenditure and GDP growth is positive and statistically significant among sampled middle-income countries (51 per unit-growth in GDP) and high-income countries (50 per unit-growth in GDP). In contrast with previous research that has found that income elasticity of health spending in middle-income countries is larger than in high-income countries, our findings show that elasticity estimates can change if different criteria are used to assemble a more homogenous group of middle-income countries. Financial protection differences between middle- and high-income countries, however, are not associated with their respective income elasticity of health spending.

**Conclusion:** The study findings show that in spite of the rapid economic growth experienced by the sampled middleincome countries, the aggregated income elasticity of health expenditure in them is less than one, and equals that of high-income countries.

## Background

As individuals become more affluent, they spend more on healthcare.^1^ A similar relationship exists across countries. High-income countries spend a higher share of their aggregate and per-capita incomes on healthcare compared to middle-income countries.^2^ A clear distinction between high- and middle-income countries, however, has become more ambiguous in recent years.^3,4^ In the last two decades many middle-income countries have been experiencing accelerated economic growth, rapid urbanization, social modernization, poverty reduction and changes in the composition of their national economies. In 1980, low- and middle-income countries accounted for 33.7% of global income whereas in 2010, they accounted for 43.4% of global income and for most of the world’s economic growth.^5^

As middle-income countries become more affluent, economically sophisticated and productive, health expenditure patterns are likely to change. Other socio-demographic and political changes that accompany rapid economic growth are also likely to influence health spending, such as government effectiveness, regulatory quality, voice and accountability.^6,7^ Population ageing is a global phenomenon that is expected to occur particularly rapidly in some middle-income countries, predominantly in China and many Eastern European and Latin American countries^8^ while other middle-income countries such as India and Nigeria would continue to have higher share of young productive populations. In addition, economic reforms in many middle-income countries have been paired with social policies that procure better investments in human capita, such as conditional cash transfers programs, micro-credits and social health insurance programs.^9,10^ Economic prosperity is often times associated with increased governmental spending on healthcare and development of private health insurance markets to cater to emerging middle classes.^11^

An extensive literature has investigated the link between economic growth and health spending to explain cross-country variation across high-income countries in the Organization for Economic Cooperation and Development (OECD) economies.^12^ This literature concludes that aggregate national income is the most important factor explaining cross-country variation in per-capita healthcare expenditure among the high-income countries of the OECD.^13,14^ A substantial share of health expenditure heterogeneity across these countries, however, remains unexplained due to data challenges.^15,16^

During the 1980s and 1990s many studies found the income elasticity of healthcare expenditures^ [1]^ in high-income countries to be more than one, which implies that healthcare expenditures rise faster than incomes. These studies supported the argument that as economies grow, healthcare becomes a superior good rather than a normal good (which would have an elasticity less than one). However, this debate is far from settled due to data and methodological concerns. Many of these studies ignored the non-stationarity among variables leading to potentially spurious results from OLS regressions.^14^ Subsequent studies which utilized advances in statistical methods have accounted for non-stationarity and autocorrelation of panel data and have found elasticities lower than one among high-income countries of the OECD indicating that healthcare remains a normal good.^15^

These analyses have recently been applied to the changing healthcare expenditure patterns of countries in different stages of economic development. Recent studies have implemented an income elasticity of health spending comparison between high-, middle- and low-income countries.^6,7^ They have investigated the role of institutional factors such as government effectiveness and accountability to explain differences across countries.^6^ These studies have found that the income elasticity of health spending is still less than one, although it is higher among middle-income countries compared to that in high-income countries.^6^

As countries develop, they are more likely to provide better financial protection from the consequences of health events through better health insurance mechanisms.^17^ High-income countries have more resources to provide better financial protection through more comprehensive public health insurance plans and a multiplicity of supplemental private health insurance plans. In contrast, public health insurance coverage in middle-income countries could be less generous, and supplemental private health insurance unavailable or inadequate. Thus, individuals in these countries may be more likely to pay for healthcare expenditures out-of-pocket (OOP) in the private sector. No study to our knowledge has investigated the role of financial protection in explaining differences in the income elasticity of health spending in middle- and high-income countries.

Implementing such a comparison is challenging, as country heterogeneity among middle-income countries is even more pronounced than heterogeneity across high-income countries. Studies advocate for alternative approaches to classifying countries based on cluster analysis.^3,4^ This study follows these recommendations, conducting a comparison in a subset of middle-income countries with large economies, populations and global influence.

### Differences Between Middle-Income and High-Income Countries

Middle-income countries are expected to experience increases in healthcare expenditures with growth in their gross domestic products* (*GDPs*)* as populations in these countries age and become more affluent. However, the trajectory of healthcare expenditure growth in middle-income countries may be different compared to high-income countries.^18^ The coexistence of lifestyle related diseases together with an epidemiological profile comparable to that of low-income countries dictates the needs that health systems have to address. Middle-income economies are currently experiencing a rapid growth of non-communicable diseases such as type 2 diabetes and heart disease, while they still have to address communicable diseases such as malaria, cholera or tuberculosis. It is important to understand the trajectory of healthcare spending compared to GDP growth in order to understand the preparedness of health systems in middle-income countries and their resource allocation patterns to different types of health services.^7^

This study aims to implement a comparison between middle- and high-income economies. This comparison takes into account the methodological recommendations from the previous literature. We collect a sample of 27 middle-income countries from Eastern Europe, Africa, Asia, the Middle East and Latin America that fulfills a set of economic and political criteria to construct a relatively homogenous sample of middle-income countries. Likewise, we select the group of 24 high-income economies from the OECD group of countries to estimate the updated per-capita health spending elasticity and compare it with the estimated elasticity in the sampled middle-income countries. This comparison serves two purposes. First, as high-income countries are extensively studied we aim to show that our methodology can produce results consistent with the existing literature for those countries.^11,18,19^ Second, we compare the elasticities in these two groups to explore whether increases in per capita wealth in middle-income countries produce expenditure responses comparable to those of high-income countries.

We hypothesize that the aggregated income elasticity of health expenditures in middle-income countries would be less than one (meaning healthcare is a normal good), supporting findings from the previous literature, but would still be larger than that of high-income countries.^6,7^ Our reasoning is that with rapid economic modernization, such as accelerated income growth and urbanization, middle-income countries face quick changes in their epidemiological profile and consumption patterns that accelerate the growth of healthcare spending. While most middle-income countries still have higher OOP costs and larger populations, their governments have also been increasingly investing in healthcare services and new medical technologies.^20^ In addition, larger segments of the population in emerging countries still lack adequate access to healthcare safety nets, which can translate into higher OOP payments to new healthcare providers.^21^

By contrast, universal healthcare and well-developed insurance markets make healthcare less substitutable in high-income countries, leading to lower elasticities. Thus, we explore whether financial protection could partially explain this difference. Financial protection is a metric that has been used to evaluate whether insured individuals are effectively covered from the financial consequences of an adverse health event. The most widely used measure of financial protection in the literature is OOP healthcare spending.^17,22^ Annual OOP health expenditures have been used to evaluate whether new public health insurance programs have been effective at reducing the uncertainty of future health spending.^22^ Cross-country comparative research on financial protection has estimated the incidence of catastrophic health spending at the country level.^21,23^ Also, economies of scale associated to universal health insurance coverage in high-income countries could translate into higher savings compared to healthcare financing in middle-income countries.

## Methods

### Sample Selection

To select a relatively homogeneous sample of middle-income countries for the analyses, three mutually exclusive political and economic criteria are used: (*i*) *Global influence*: middle-income countries with G-20 membership, (*ii*) *Rapid economic transformation*: be part of the Dow-Jones and N-11 lists of emerging markets,^24,25^ (*iii*) *Large internal markets*: be classified as an “Emerging and Developing Country” by the IMF, have a population of at least 5 million people and a national GDP of at least $130 000 million in 2014.^26^ The 27 middle-income countries from Africa, the Middle East, South and East Asia, Eastern Europe and Latin America that fulfill at least two of the three criteria are listed in Table 1.

**Table 1:** Economic Indicators Across Sampled Middle-Income Economies (1995-2014)^a^

**Country**	**GDP Per Capita PPP**	**Per Capita HE**	**HE as % of GDP**	**Government HE as % of Total HE**	**OOP Expenditure as % of Total HE**	**Percent 65 and Over**	**Life Expectancy**	**Infant Mortality Rate (deaths/1000 Live Births)**
Algeria	10 435	475	4.34	72.56	26.47	4.59	73.42	51.00
Argentina	15 078	1049	7.31	56.83	27.74	10.45	75.34	51.20
Brazil	11 521	893	7.60	43.50	33.69	5.98	70.75	96.90
Chile	15 204	1032	6.64	46.31	41.70	7.98	76.56	51.20
China	5899	288	4.65	45.72	47.02	7.66	72.67	84.50
Colombia	8832	599	6.81	73.16	18.81	5.36	72.66	28.00
Czech Republic	21 926	1534	6.90	87.43	11.80	14.63	75.89	1.20
Egypt	8367	429	5.07	40.13	57.15	3.93	70.62	142.90
Hungary	18 267	1386	7.53	70.42	24.64	15.69	72.79	1.90
India	3138	136	4.29	26.23	66.40	4.85	64.08	36.10
Indonesia	6574	171	2.50	36.83	47.29	5.29	69.25	30.50
Iran	12 934	775	5.68	41.44	52.53	4.72	67.97	73.25
Malaysia	16 478	595	3.51	56.07	33.66	4.38	72.03	95.20
Mexico	13 252	767	5.69	46.08	50.78	5.41	74.67	106.85
Nigeria	3617	131	3.53	28.53	67.78	2.89	48.21	133.30
Pakistan	3477	99	2.83	26.91	63.59	4.06	63.55	87.90
Peru	7383	361	4.82	55.55	36.49	5.48	70.34	160.90
Philippines	4437	173	3.76	38.23	50.59	3.77	68.96	151.20
Poland	15 830	1001	6.19	70.59	25.96	13.00	74.72	66.50
Romania	12 660	644	4.86	79.53	20.10	14.03	72.06	97.35
Russia	16 976	1060	6.12	60.44	32.67	13.09	67.27	50.80
Saudi Arabia	40 359	1504	3.71	67.98	20.46	2.71	72.73	97.00
South Africa	9769	809	8.27	44.00	10.85	4.67	55.33	129.45
Thailand	10 328	392	3.75	65.57	24.56	7.35	71.89	55.85
Turkey	13 262	683	4.97	72.02	21.70	5.82	70.84	82.55
UAE	74 483	2113	2.91	69.88	21.37	0.85	75.07	1.50
Venezuela	14 101	694	4.91	39.15	54.68	5.04	72.95	86.90
**Total (N = 27)**	14 614	733	5.15	54.11	36.68	6.81	70.09	76.00
**SD**	14 456	551	1.73	17.54	17.41	3.99	6.47	56.97
**Min**	1522	52	1.93	19.59	6.49	0.76	44.00	1.00
**Max**	92 863	2454	9.38	90.89	75.23	17.83	78.40	172.00
**No. of observations**	540	540	540	540	540	539^b^	539^b^	540

Abbreviations: GDP, gross domestic product; HE, health expenditure; PPP, purchasing-power-parity; OOP, out-of-pocket. ^a^ Mean values between 1995-2014. Data on country HE, public HE and OOP spending are from WHO sources. Data on GDP growth is from the IMF. Share of elderly population is from the US Census international data. ^b^ Data missing for Egypt for 1995.

Middle-income countries face different trajectories of health spending growth and development of healthcare markets compared to those of high-income countries. Hence, we compare the patterns of growth of healthcare spending with GDP in these emerging countries to those of 24 high-income countries of the OECD in order to discern potentially different patterns of growth in healthcare spending.

### Data Sources

The analyses utilize data from 27 emerging countries and 24 high-income countries to compare their aggregate elasticity over a period of 20 years from 1995-2014. The data sources include the World Health Organization (WHO), the International Monetary Fund (IMF) and the US Census Bureau’s International data.^27,28^ Data on country health expenditure, public health expenditure and OOP spending are from WHO sources. Data on per-capita GDP is from the IMF. These measures are merged with international data from the US Census to estimate the share of the older adult (65 and above) population in each country, and their life expectancy.^29^

### Health Spending

This study uses the econometric framework from the previous literature on the cross-country relationship between health expenditure and income.^6,7^ Consequently, the natural log of per-capita healthcare expenditures (health expenditure, subsequently) is the dependent variable in this study. It is estimated in terms of purchasing-power-parity (PPP) and expressed in current international dollars.^28^

### Independent Variables

The main explanatory variable corresponds to the natural log of GDP per-capita (income, hereafter) estimated in PPP and expressed in international dollars.^27^ Additional variables (identified by previous literature as important determinants of cross-country differences in health expenditures) included in the analyses are the following: (*i*) the share of government health expenditure as a percentage of total health expenditure (government expenditures, subsequently) to capture any shifts in government financing of the health sector, (*ii*) the share of OOP health spending to investigate the role of financial protection, (*iii*) the percentage of the older adult population who are 65 years of age or older, and the percentage of children under 5 years of age, to control for heterogeneous demographic characteristics across countries, and to account for variation in demand for healthcare due to population patterns, and (*iv*) average life expectancy and infant mortality to account for supply-side factors. All continuous variables in the empirical analyses are included in natural logarithm form in the regression model. We conduct several sensitivity analyses, which are included in the statistical appendix.

### Statistical Analyses

All statistical analyses are conducted using Stata 14. The data are stratified by their status as middle- and high-income countries for a side-by-side comparison. All variables are included in their natural logarithm forms. All tests and regressions are repeated for middle- and high-income countries separately. The possibility of non-stationarity and cointegration of income, health expenditures and related explanatory variables in high-income countries has been stressed in the literature.^12^ Based on the results of unit root tests and test for serial correlation (described in more detail below), we implement the first-difference of study variables to de-trend the panel data,^30^ following prior literature.^6,7^ Further tests for unit roots and serial correlation confirm that using first differenced variables would address issues of unit roots within the model. We utilize Hausman and Breusch-Pagan Lagrange multiplier tests to determine that a fixed effects model with time fixed-effects is the best model compared to a random effects model (statistical appendix).

Our regression approach can be summarized by the following regression equation:

Δy_it_ = α_i_ + Δβ_i_X_it_ + δ_t_ + u_it_

where *y*_it_ represents health expenditures across countries (*i = 1,2,3…27 for middle-income countries and i=1,2..24 for high-income countries*) and across time (*t = 1,2,3…20*), the term *X*_it_ includes income and all additional explanatory variables described above. The term *α*_i_ is the intercept for each country and *δ*_t_ is the intercept for time fixed-effects. The term *u*_it_ represents the error term.

## Results

### Descriptive Statistics

Tables 1 and 2 show the mean values for each middle- and high-income country respectively. Over the study period (1997-2014), the UAE and Saudi Arabia reported the highest GDP growth rates whereas Russia and Romania had the lowest growth. Average per capita GDP was the highest in UAE and Saudi Arabia, while Nigeria, India and Pakistan reported the lowest values. In the case of high-income countries, Israel and Ireland reported the highest average growth rates and Germany and Japan had the lowest growth rates during the study period. Portugal, South Korea and Israel had the lowest GDP per capita and Luxembourg, Norway and Switzerland had the highest GDP per capita.

**Table 2:** Economic Indicators across Large High-Income Economies (1995-2014)

**Country**	**GDP Per Capita PPP**	**Per Capita HE**	**HE as % of GDP**	**Government HE as % of Total HE**	**OOP Expenditure as % of Total HE**	**Percent 65 and Over**	**Life Expectancy**	**Infant Mortality Rate (deaths/1000 Live Births)**
Australia	34 807	2986	8.44	67.03	18.33	13.01	80.55	4.25
Austria	36 203	3823	10.46	74.92	16.15	16.59	79.03	1.10
Belgium	34 091	3177	9.14	76.23	19.21	17.08	78.62	1.45
Canada	34 504	3401	9.71	70.42	15.00	14.28	80.50	47.00
Finland	32 587	2749	8.31	73.50	20.34	16.18	78.44	1.00
France	32 521	3465	10.56	78.36	7.10	16.29	80.01	72.35
Germany	34 672	3663	10.48	77.88	12.62	18.41	78.81	67.25
Greece	24 238	2152	8.77	60.20	35.21	18.31	78.99	1.60
Iceland	33 199	2976	8.95	81.68	17.16	11.96	80.47	1.00
Ireland	37 934	2846	7.30	72.93	15.85	11.30	78.45	1.00
Israel	24 149	1805	7.45	63.04	26.52	9.87	79.88	2.00
Italy	31 477	2683	8.43	74.21	22.84	19.22	80.52	50.00
Japan	29 640	2519	8.32	81.57	15.09	19.74	82.07	77.40
Luxembourg	74 026	5327	7.08	86.84	9.96	14.56	78.94	1.00
The Netherlands	38 143	3527	9.01	74.91	7.12	14.60	79.26	2.00
New Zealand	26 486	2441	8.89	79.92	13.77	12.37	79.28	1.00
Norway	53 855	4827	8.91	83.82	15.39	15.26	79.48	1.00
Portugal	22 703	2119	9.20	67.26	23.62	16.79	77.22	1.40
South Korea	22 655	1316	5.41	51.48	39.09	9.04	77.77	56.70
Spain	27 761	2315	8.21	72.51	22.16	16.73	79.99	45.80
Sweden	35 083	3385	9.41	83.35	14.90	17.87	80.42	1.00
Switzerland	45 145	4816	10.56	59.65	29.87	15.90	80.86	1.00
UK	31 053	2567	8.06	81.56	10.26	16.07	78.70	93.80
USA	42 062	6444	15.04	45.39	13.20	12.83	77.57	63.65
**Total (N = 24)**	34 958	3222	9.00	72.44	18.36	15.19	79.41	24.87
**SD**	13 085	1492	1.98	10.71	8.19	3.08	1.76	32.91
**Min**	12 079	444	3.67	37.53	5.22	5.82	73.60	1.00
**Max**	96 035	9318	17.14	92.81	53.13	25.77	84.50	120.00
**Observations**	480	480	480	480	480	479^a^	479^a^	479^a^

Abbreviations: GDP, gross domestic product; HE, health expenditure; PPP, purchasing-power-parity; OOP, out-of-pocket. ^a^ Data missing for Ireland for 1995.

The sampled middle-income countries that allocated a higher share of GDP to healthcare were South Africa, Brazil and Hungary, while Indonesia, UAE and Pakistan allocated the lowest. In the case of per-capita health spending, the Czech Republic, UAE and Saudi Arabia reported the highest expenditures, compared to Pakistan, Nigeria and India that reported the lowest. In the case of high-income countries, South Korea, Luxembourg and Ireland reported the lowest share of health spending as a share of GDP whereas the United States, Switzerland and France reported the highest. In the case of per capita health spending, the United States, Luxembourg and Norway had the highest values and South Korea, Israel and Portugal reported the lowest.

From the financial protection perspective, among middle-income countries, Nigeria, Pakistan and Egypt reported the highest OOP share of health spending, while Romania, Saudi Arabia and South Africa reported the lowest. In the case of high-income countries, South Korea, Greece and Switzerland reported the highest share of OOP spending from total health expenditures while Luxemburg, the Netherlands and France reported the lowest.

Eastern European countries in the sample (Hungary, Czech Republic, Romania, Poland and Russia) had the highest shares of the elderly population (65 and over), while Nigeria, Saudi Arabia and the UAE had the lowest share. These data trends suggest a more advanced demographic transition in Eastern Europe. In the case of high-income countries, Italy, Japan and Germany had the highest shares of the older adult population and South Korea, Israel and Ireland had the lowest share of older adults.

### Model Selection

Detailed results of the model fit and other tests described in this section are provided in the statistical appendix. First, we conducted a series of unit root tests to check for stationarity in variables with logarithmic raw form or by de-trending them. Non-significant results from these tests indicated that the presence of unit root could not be rejected. Then, we tested the first differenced data for the same specifications. The significant results with the first differenced variables indicated absence of unit roots and hence, we used first differenced variables for the rest of the analyses.

Although our preferred regression model based on earlier literature is the fixed effects model, we conducted Hausman tests to confirm this choice by comparing fixed and random effects models, with a single covariate (first differenced logarithm of per capita GDP) and then again with all covariates. Results from these tests indicate that a fixed effects model was a better fit for the data. We confirmed these results with Breusch-Pagan Lagrange multiplier tests, where non-significant *P* values indicate that ordinary least squares (OLS) with year fixed effects is a better fit compared to the corresponding random effects models.

We concluded this section of the analyses conducting the Pesaran test for cross-sectional dependence (CSD) and contemporaneous correlation and Woolridge test for autocorrelation. The non-significant F-values from these tests indicate that our preferred models do not have CSD or autocorrelation issues (details are provided in the statistical appendix).

### Elasticities

Table 3 summarizes the main panel regression analyses with robust standard errors. The reduced models estimate the basic relationship between health expenditures and GDP growth, without year fixed-effects or additional covariates. The results confirm a positive relationship between income and health spending with an aggregated income elasticity of health spending less than one. When time fixed-effects and all the explanatory variables are fitted into the full model, effect sizes change from 0.49 to 0.51 for the middle-income countries and from 0.55 to 0.50 for high-income economies. All these coefficients are statistically significant. Comparisons of the coefficients across the models indicate that the coefficients are statistically different. In addition, the aggregate elasticity is less than one. The magnitude of the estimated aggregate income elasticity of health spending for emerging countries is essentially identical to the one in high-income economies. Most explanatory variables are not statistically significant in the full models, including OOP spending. The only exceptions were growth in government spending and in the share of the older adult population that were statistically significant in the case of high-income countries.

**Table 3:** Panel Analysis: Determinants of Per Capita Healthcare Expenditure

**∆Log HE as % of GDP**	**Middle-Income Countries**		**High-Income Countries**	
	**Reduced Model**	**Full Model**	**Reduced Model**	**Full Model**
∆Log GDP per capita	0.49***	0.51***	0.55***	0.50***
	(0.10)	(0.10)	(0.09)	(0.11)
∆Log government HE as % of total HE		0.05		0.30*
		(0.14)		(0.22)
∆Log OOP. HE as % of total HE		-0.06		0.07
		(0.08)		(0.15)
∆Log life expectancy		0.32		0.30
		(0.27)		(0.65)
∆Log infant mortality		-0.01		0.00
		(0.01)		(0.00)
∆Log Pop. >65		0.03		-0.55*
		(0.36)		(0.24)
∆Log Pop. <5		0.14		-0.11
		(0.18)		(0.15)
Constant	0.04***	0.03***	0.03***	0.04***
	(0.00)	(0.01)	(0.00)	(0.01)
N	513	512	456	455
No. of countries	27	27	24	24
*t* (years)	20	20	20	20
R-squared	0.06	0.07	0.14	0.16
AIC	-1177.70	-1171.50	-1695.79	-1693.26
BIC	-1173.46	-1146.07	-1691.67	-1668.53

Abbreviations: GDP, gross domestic product; HE, health expenditure; OOP, out-of-pocket; AIC, Akaike Information Criterion; BIC, Bayesian Information Criterion. * *P* < .05; ** *P* < .01; *** *P* < .001. Robust standard errors are in parentheses.

Following the previous literature, we considered including variables that represent governance factors, voice and accountability and regulatory control over the same period.^6^ However, many emerging countries included in our study do not have these measures reported consistently leading to a very small sample size. Hence, we do not include those variables in our analyses although they influence the cost, quality, outcomes and effectiveness of healthcare.

## Discussion

As countries develop and become more affluent, spending priorities of households and governments change. The literature on the cross-country relationship between health expenditure and income in high-income countries has investigated whether healthcare can be considered a superior good (elasticity >1) or a normal good (elasticity <1). In other words, the question is whether countries spend more, the same or less on healthcare as they become richer. This is an important issue for middle-income economies as their health systems are trying to keep pace with their rapid economic development. Knowing how economic growth affects health spending can provide useful information to government planners and international organizations. In addition, comparing the respective elasticities of middle-income countries with those of currently high-income countries can provide new insights about possible consequences of government expenditures, financial protection, and growing shares of older populations across different stages of economic development.

This study is among the first studies that extend the analysis of income elasticity of health spending to middle-income countries.^6,7^ One of the differences between previous studies and our study is the selection of countries included in the analysis. While previous studies include more heterogeneous set of countries in broad income categories such as middle- or low-income countries as defined by World Bank, our data is more homogenous and representative of a subset of middle-income countries, which were chosen based on economic, political and demographic criteria described earlier. Our estimates use the latest econometric techniques that have been implemented in recent studies to address methodological issues such as serial-correlation and co-integration that are inherently present in time-series data.^6,7^

While previous research has used a comprehensive sample of middle-income countries, we have followed the advice from the recent literature that recommends conducting cross-country comparison in more homogenous subsets of countries.^3,4^ In this study we focus on emerging countries with large populations, economies and global influence. In contrast to the previous findings, our estimates of income elasticity of health spending are nearly identical to those of high-income countries. This finding could possibly suggest the convergence of health spending and income growth dynamics between sampled middle- and high-income countries.

In addition, our comparison looks at the share of OOP spending as a percentage of health spending to examine the potential role of financial protection in explaining differences in the income elasticity of health spending between middle- and high-income countries. More fragmented coverage and less regulated healthcare markets in middle-income countries could suggest less financial protection and a higher share of income growth allocated to health spending. In high-income countries, governments are the main financers and sometimes even the main providers of healthcare. By contrast, the size of the private delivery system that is financed through OOP spending is larger in middle-income countries.^2^ In contrast with high-income economies where private insurers negotiate payments with private providers, private health insurance markets in middle-income countries are still in their initial formative stages.

With a few exceptions, most countries in our sample of middle-income countries have fragmented health systems that offer different types of coverage to different populations. In contrast with most high-income countries where universal healthcare or near universal healthcare coverage takes full advantage of economies of scale, the fragmentation of health systems in many middle-income countries may increase administration costs that ultimately are reflected in higher overall health expenditures as incomes rise. According to our findings, however, the OOP share of health spending is non-statistically significant in both middle- and high-income countries. Future research should investigate whether this relationship holds over time as healthcare funders in both sets of countries reduce costs partly by increasing the cost sharing among healthcare users.

Our study identified a positive and small but statistically significant relationship between growth in government expenditures and the aggregate income elasticity of health expenditure in high-income countries. This association may be suggestive of the increasing role of government financing in the health sector of this group of countries. In 2014, the governments of high-income countries were responsible for approximately 65% of national health expenditures.^31^ By contrast, the 27 governments of emerging countries analyzed in this study were responsible for approximately 35% of national health expenditures.^28^ If governments in middle-income countries continue to increase their financing of the health sector and eventually reach the share observed in high-income countries, it is uncertain whether the income elasticity of health spending would go any lower.

As populations grow older, they are expected to spend more on healthcare as a consequence of increased utilization by the older adult population. However, this study did not find any evidence that growth in the share of the elderly population is related to health expenditure growth among the sampled group of middle-income countries. Growth in population ageing was negative and statistically significant in high-income countries only. These results may suggest a weak influence of these factors in driving overall health expenditure growth. Future research could explore whether related independent variables that could impact health spending, such as the availability of new medical technologies, are significantly associated with the income elasticity of health spending once population ageing accelerates in middle- and high-income countries.^20^

Middle-income countries can benefit from shared knowledge on how to pair accelerated economic development with more effective, efficient and fair healthcare systems. The results of this study also suggest that government planners in the sampled middle-income countries should still find ways to pair income growth with rapidly changing health systems where health-spending evolution may differ from that of current patterns among high-income countries.

## Study Limitations

While this analysis is informative on the aggregate level of health spending growth and income, some limitations should be acknowledged. Country-level results are difficult to examine in regards to the interaction of income and health expenditure growth without accounting for other less aggregate measures such as income inequality, quality of care, changing health status and increasing education and living standards.^11,32^ Non-linear and changing relationships across countries should be further investigated.^18,19^ While data sources for most countries in this study are reliable and results remain robust to different specifications, cross-country differences do exist in data recording standards and methodologies and hence, the results should be interpreted with caution.

## Conclusion

This study shows that health expenditures in emerging countries can still be considered a normal good rather than a superior good. Our point estimates suggest that the income elasticity of health spending is similar in middle- and high-income countries. Our findings contrast with previous research that used a broader sample of middle-income countries. It is currently uncertain whether health expenditure dynamics will change as middle-income countries transition towards universal health coverage and their healthcare markets become more sophisticated. We find no evidence that differences in financial protection and population ageing growth rates may take them in a different trajectory as the one experienced by current high-income countries.

## Ethical issues

Secondary data analyses from publicly available sources are not subject to IRB review at UCLA or CSULB.

## Competing interests

Authors declare that they have no competing interests.

## Authors’ contributions

AVB: study conception, research design, data analysis and interpretation, and manuscript editing. SVS: data analysis and manuscript editing.

## Authors’ affiliations

^1^Department of Health Policy and Management, Fielding School of Public Health, University of California, Los Angeles, CA, USA. ^2^Department of Health Care Administration, California State University, Long Beach, CA, USA.

## Endnotes


[1] Income elasticity is defined as the percentage change in healthcare expenditure associated with 1% change in income.


## Key messages

**Implications for policy makers**

- Middle-income countries can benefit from shared knowledge on how to pair economic growth with more effective, efficient and fair healthcare systems.
- Government planners in middle-income countries would benefit from a better understanding of how their economic and health system development compares with those of high-income nations.

**Implications for public**

If present accelerated growth rates in middle-income countries continue, they are likely to have an increasing role in the world’s healthcare financing. This study shows middle-income countries allocate less money to healthcare, but as their economies grow, their health spending follows a trajectory similar to that in high-income countries. This study looks at the tradeoff between economic growth and health spending growth and finds that economic growth still increases faster than health spending. The rate of growth of health spending is also similar among sampled middle-income countries. Government planners should observe these trends carefully to identify whether health spending growth rates will follow a similar trajectory to those of high-income nations or if health spending in middle-income countries would evolve differently.

## Statistical Appendix

**1.** Lagged Unit Root Tests

**Lagged Variables**									
	**Without Trend**					**With Trend**			
	**3ADF**	**2ADF**	**1ADF**	**0ADF**		**3ADF**	**2ADF**	**1ADF**	**0ADF**
Log GDP per capita	0.95	1.94	0.92	-0.60		-0.19	0.38	-2.64**	7.72
Log HE as % of GDP	-0.14	1.22	2.75	4.00		5.20	2.87	1.58	3.31
Log Pop. >65	5.04	4.86	5.30	4.78		5.64	5.94	7.16	9.14
Log % of Govt. HE	0.65	0.11	-1.49	0.01		3.22	2.90	1.97	2.47
Log OOP as % of private HE	-1.06	-1.88*	-1.70*	-1.00		1.82	0.64	0.92	2.39
Log Life Exp.	-	-	-	-		-	-	-	-
Log infant mortality	-	-	-	-		-	-	-	-
Log Gini coefficient	-	-	-	-		-	-	-	-
**First Differenced Lagged Variables**									
	**Without Trend**					**With Trend**			
	**3ADF**	**2ADF**	**1ADF**	**0ADF**		**3ADF**	**2ADF**	**1ADF**	**0ADF**
∆Log GDP per capita	-3.15**	-3.72**	-6.53**	-4.52**		-1.90*	-1.77*	-4.62**	-3.00**
∆Log HE as % of GDP	0.88	-3.05**	-4.14**	-8.21**		-0.81	-3.07**	-2.28*	-6.8**
∆Log Pop. >65	2.57	2.25	2.32	-1.88*		2.78	2.11	2.23	-3.44**
∆Log % of Govt. HE	-0.84	-1.64*	-4.74**	-10.74**		-2.67*	-0.81	-3.60**	-9.64**
∆Log OOP as % of private HE	-1.58*	-3.97**	-4.89**	-10.53**		-0.73	-3.35**	-3.48**	-9.25**
∆Log Life Exp.	-4.34**	-3.26**	-6.24**	-16.47**		0.00**	-2.02*	-5.12**	-15.55**
∆Log infant mortality	-8.87**	-4.86**	-10.74**	-19.96**		-7.54**	-3.13**	-10.24**	-17.62**
∆Log Gini coefficient	-	-	-	-		-	-	-	-

Abbreviations: GDP, gross domestic product; HE, health expenditure; OOP, out-of-pocket; ADF, augmented Dickey-Fuller Regressions.

**2.** Model Fit Tests

**Hausman test to test between fixed and random effects models – significant** ***P*** ** value indicates FE model is preferred**							
			FE	RE	Chi2	*P* value	Preferred Model
Middle-income countries	Simple model - One covariate	fd_ln_gdp_pc	0.4881235	0.5892946	7.58	0.0059	FE
High-income countries	Simple model - One covariate	fd_ln_gdp_pc	0.5467499	0.6034603	10.66	0.0011	FE
Middle-income countries	Multiple covariates	fd_ln_gdp_pc	0.5027975	0.5949999	8.69	0.1918	-
High-income countries	Multiple covariates	fd_ln_gdp_pc	0.5006531	0.5594028	16.16	0.0129	FE
**Breusch-Pagan Lagrange multiplier tests for random effects – a non-significant** ***P*** ** value indicates that OLS is preferred to RE.**							
**As OLS with dummy years is similar to FE, we take it to indicate that FE is preferred to RE from this test. **							
Middle-income countries				Chi2	*P* Value	Preferred Model	
High-income countries	Multiple covariates	fd_ln_gdp_pc	0.5949999	0	1	OLS	
	Multiple covariates	fd_ln_gdp_pc	0.5594028	0	1	OLS	
**Pesaran test for CSD and contemporaneous correlation – non-significant results indicate no CSD**							
Middle-income countries				Chi2	*P* Value	Preferred Model	
High-income countries	Multiple covariates	fd_ln_gdp_pc	0.5949999	0	1	No CSD	
	Multiple covariates	fd_ln_gdp_pc	0.5594028	0	1	No CSD	
**Woolridge test for autocorrelation in panel data – non-significant results indicate no CSD**							
Middle-income countries				*F* Value	*P* Value	Preferred Model	
High-income countries	Multiple covariates	fd_ln_gdp_pc	0.7351903	1.129	0.2977	no autocorrelation	
	Multiple covariates	fd_ln_gdp_pc	0.4800522	0.036	0.8518	no autocorrelation	

Abbreviations: CSD, cross sectional dependence; FE, fixed effects; OLS, ordinary least squares.
